# Subtype-specific differences in Gag-protease replication capacity of HIV-1 isolates from East and West Africa

**DOI:** 10.1186/s12977-021-00554-4

**Published:** 2021-05-05

**Authors:** Omotayo Farinre, Kamini Gounder, Tarylee Reddy, Marcel Tongo, Jonathan Hare, Beth Chaplin, Jill Gilmour, Phyllis Kanki, Jaclyn K. Mann, Thumbi Ndung’u

**Affiliations:** 1 HIV Pathogenesis Programme, The Doris Duke Medical Research Institute, University of KwaZulu-Natal, Durban, South Africa; 2 Africa Health Research Institute, Durban, 4001 South Africa; 3 Biostatistics Research Unit, South African Medical Research Council, Durban, South Africa; 4 Centre of Research for Emerging and Re-Emerging Diseases (CREMER), Yaoundé, Cameroon; 5 International AIDS Vaccine Initiative (IAVI) Human Immunology Laboratory (HIL), Imperial College, London, UK; 6 IAVI Global Headquarters, 125 Broad Street, 9th Floor,, New York, NY USA; 7 Department of Immunology and Infectious Diseases, Harvard T.H. Chan School of Public Health, Boston, MA USA; 8 Ragon Institute of Massachusetts General Hospital, Massachusetts Institute of Technology and Harvard University, Cambridge, MA USA; 9 Max Planck Institute for Infection Biology, Berlin, Germany; 10 Division of Infection and Immunity, University College London, London, UK

**Keywords:** HIV-1 subtype, HIV-1 recombinants, Sub-Saharan Africa, HIV-1 replication capacity, HLA class I alleles

## Abstract

**Background:**

The HIV-1 epidemic in sub-Saharan Africa is heterogeneous with diverse unevenly distributed subtypes and regional differences in prevalence. Subtype-specific differences in disease progression rate and transmission efficiency have been reported, but the underlying biological mechanisms have not been fully characterized. Here, we tested the hypothesis that the subtypes prevalent in the East Africa, where adult prevalence rate is higher, have lower viral replication capacity (VRC) than their West African counterparts where adult prevalence rates are lower.

**Results:**

*Gag-protease* sequencing was performed on 213 and 160 antiretroviral-naïve chronically infected participants from West and East Africa respectively and bioinformatic tools were used to infer subtypes and recombination patterns. VRC of patient-derived *gag-protease* chimeric viruses from West (n = 178) and East (n = 114) Africa were determined using a green fluorescent protein reporter-based cell assay. Subtype and regional differences in VRC and amino acid variants impacting VRC were identified by statistical methods. CRF02_AG (65%, n = 139), other recombinants (14%, n = 30) and pure subtypes (21%, n = 44) were identified in West Africa. Subtypes A1 (64%, n = 103), D (22%, n = 35), or recombinants (14%, n = 22) were identified in East Africa. Viruses from West Africa had significantly higher VRC compared to those from East Africa (*p* < 0.0001), with subtype-specific differences found among strains within West and East Africa (*p* < 0.0001). Recombination patterns showed a preference for subtypes D, G or J rather than subtype A in the p6 region of *gag*, with evidence that subtype-specific differences in this region impact VRC. Furthermore, the Gag A83V polymorphism was associated with reduced VRC in CRF02_AG. HLA-A*23:01 (*p* = 0.0014) and HLA-C*07:01 (*p* = 0.002) were associated with lower VRC in subtype A infected individuals from East Africa.

**Conclusions:**

Although prevalent viruses from West Africa displayed higher VRC than those from East Africa consistent with the hypothesis that lower VRC is associated with higher population prevalence, the predominant CRF02_AG strain in West Africa displayed higher VRC than other prevalent strains suggesting that VRC alone does not explain population prevalence. The study identified viral and host genetic determinants of virus replication capacity for HIV-1 CRF02_AG and subtype A respectively, which may have relevance for vaccine strategies.

**Supplementary Information:**

The online version contains supplementary material available at 10.1186/s12977-021-00554-4.

## Background

Sub-Saharan Africa bears the brunt of the HIV-1 epidemic with approximately 70% of the estimated people living with the virus globally [1]. The epidemic in sub-Saharan Africa is heterogeneous, with significant regional differences in prevalence and multiple HIV-1 subtypes that are unevenly distributed across the continent [2–4]. Specifically, West Africa is reported to have an adult HIV-1 prevalence rate of approximately 2%, although slight variations have been reported between countries [5]. CRF02_AG is a circulating recombinant form (CRF) that constitutes the most common HIV-1 strain circulating in West Africa, however, almost all known pure subtypes have been identified within the region, as well as several CRFs and unique recombinant forms (URF) [2, 5–7]. In East Africa, HIV-1 prevalence rates are higher at above 5% [8, 9], with subtypes A1, C and D and their recombinants co-circulating. Multiple factors account for the heterogeneity of HIV prevalence and disease progression rates across sub-Saharan Africa, including sociocultural factors such as male circumcision practices, socioeconomic factors, coinfections, microbiota, host genetic and viral factors [10–15]. Considering that curtailing HIV transmission and providing optimal treatment remain formidable public health challenges worldwide, a better understanding of the viral factors that enhance transmission efficiency or the rate of disease progression following infection may be required for novel interventions such as vaccines [16].

It is well documented that HIV-1 subtypes display differences in the rate of disease progression. For example, in West Africa, individuals infected with non-A subtypes were more likely to develop AIDS compared to those with subtype A, whereas infection with the recombinant HIV-1 A3/CRF02_AG was associated with increased risk of AIDS and AIDS-related death compared to the sub-subtype A3 [17, 18]. In East Africa, HIV-1 subtype D has been associated with faster disease progression than subtype A, whereas in southern Africa, subtype C was associated with significantly slower rates of CD4 T-cell decline and higher frequencies of long-term non-progression compared to subtypes A or D [19–22]. Interestingly, studies have suggested that the more prevalent or efficiently transmitted subtypes are not necessarily the ones associated with faster disease progression, suggesting that the biological factors that determine transmission efficiency and in vivo virulence can be uncoupled and are distinct. For example, infections with HIV-1 subtype A were associated with higher transmission rates than subtype D [20, 23], which may have resulted in the increasing prevalence of subtype A over D within the study population over a period of time [24]. Similarly, it has been proposed that a transmission-virulence evolutionary trade-off may explain the overall increasing relative prevalence of the more transmissible subtype A over the more virulent subtype D strain in Uganda; and subtype C is speculated to have become predominant in sub-Saharan Africa partly as a result of its lower virulence compared to subtypes A and D [25]. Other studies have argued that socio-historical rather than evolutionary factors have been the main determinants of the rapid expansion of subtype C in sub-Saharan Africa [26].

Overall, the viral factors that may underlie variation in transmission efficiency and virulence are poorly understood. However, subtype-specific functional differences in various HIV-1 genetic loci are now well documented, suggesting that some of these may account for the distinct transmission patterns, epidemic spread, and rates of disease progression [27–34]. Gag-protease mediated VRC has been shown to differ between HIV-1 subtypes and correlates strongly with whole virus isolates, making it an important determinant of the rate of disease progression [35–38]. We and others have also shown that HIV-1 transmission selects for consensus-like viral variants and that similarity to consensus correlates with lower VRC [39, 40]. Overall, accumulating evidence suggests that the HIV-1 transmission genetic bottleneck favors consensus-like viruses that may have lower VRC, but that increased VRC in vitro is an important determinant of a faster rate of HIV disease progression. In this study, using chronically infected antiretroviral-naïve patient samples, we sought to further examine the diversity of circulating HIV-1 strains in West and East Africa, two regions of sub-Saharan African with distinct patterns of low versus moderately high prevalence of HIV and to investigate whether there are differences in *gag-protease*-mediated VRC that may explain the reported differences in prevalence and subtype-specific rates of disease progression. Furthermore, we also wanted to identify viral genetic and host genetic determinants of differences in VRC, considering that this information may have important implications for biomedical prevention and treatment strategies against HIV.

## Materials and methods

### Study participants

This was a retrospective cross-sectional study, where ART-naive plasma samples collected between 2000 and 2010 from chronically infected individuals in West and East Africa were analyzed. In West Africa, study participants were from previously described cohorts from the Cameroon (n = 169) [6], Nigeria (n = 31) [41] and Senegal (n = 96) [42]. Age, sex, and viral load data were available for most participants from Cameroon, and some of the participants had CD4 + T cell count data. Participants from Senegal were all female and only CD4 + T cell count data were available, while no demographic or clinical information was available for participants from Nigeria. In East Africa, study participants were from the well characterized IAVI Protocol-C cohort [43] from Kenya (n = 73), Rwanda (n = 61) and Uganda (n = 107) [43]. However, samples from East Africa were preselected for subtypes A, AD and D based on previous *pol* gene sequencing [43]. Age, sex, viral load, CD4 + T cell count and HLA class I data were available for participants from East Africa.

### Viral RNA extraction

Viral RNA was extracted from chronically infected patient plasma samples using the QIAamp® Viral RNA Mini extraction kit (Qiagen, Hilden, Germany) according to the manufacturer’s protocol. Samples with a viral load of less than 5,000 copies/mL were concentrated by spinning 500 µL of plasma at 14,000 × *g* for 2 h at 4 °C. Approximately 350 µL of supernatant was removed and discarded. The pellet was then re-suspended in the remaining volume of plasma and used for RNA extraction.

### Amplification and viral sequencing

Extracted viral RNA was converted into cDNA and then double-stranded DNA by a one-step RT-PCR using the Superscript III One-Step RT-PCR, Platinum Taq High Fidelity kit (Invitrogen, San Diego, USA). Each reaction was made up of 20 µL of 2X reaction mix, 0.8 µL of each of the 10 µM forward Gag + 1 (5′-GAG GAG ATC TCT CGA CGC AGG AC-3′; HXB2 numbering 675–697) and reverse 3′RVP (5′-GGA GTG TTA TAT GGA TTT TCA GGC CCA ATT-3′; 2725–2696) primers, 0.8 µL of Superscript III RT/Platinum Taq High Fidelity Enzyme Mix and 4 µL of RNA. A second round of PCR was performed to amplify the 1,760 bp *gag-protease*, HXB2 positions 790–2550 [44] using the TaKaRa Ex Taq HS enzyme kit (Shiga, Japan). Each reaction consisted of 5 µL of 10Ex Taq Buffer, 4 µL 2.5 mM dNTP, 0.8 µl each of 10 µM forward primer HXB2 695–794 (5′-GAC TCG GCT TGC TGA AGC GCG CAC GGC AAG AGG CGA GGG GCG GCG ACT GGT GAG TAC GCC AAA AAT TTT GAC TAG CGG AGG CTA GAA GGA GAG AGA TGG G-3′), and reverse primer HXB2 2,704–2,605 (5′-GGC CCA ATT TTT GAA ATT TTT CCT TCC TTT TCC ATT TCT GTA CAA ATT TCT ACT AAT GCT TTT ATT TTT TCT TCT GTC AAT GGC CAT TGT TTA ACT TTT G-3′) that are the NL4-3 sequences immediately flanking *gag-protease*, 0.25 µL of ExTaq enzyme and 2 µL of the RT-PCR product. Amplification of *gag-protease* was confirmed by agarose gel electrophoresis and amplicons were then sequenced as previously described [35]. The resulting amplicon was diluted 1:15 in distilled water and population sequenced using the ABI PRISM Big Dye Terminator Ready reaction mix V3 (Applied Biosystems, Waltham, USA) and run on an ABI 3130xl Genetic Analyzer (Applied Biosystems, Waltham). Sequences were assembled and edited using the Sequencher 5.1 (Genecodes, USA) software program and were aligned to the HIV-1 subtype B reference strain HXB2 (GenBank accession no. K03455). Full-length *gag-protease* sequences were submitted under the accession numbers MW123927- MW124300.

### HIV-1 subtyping

Edited sequences were exported as a FASTA file and then aligned and translated using the Gene-Cutter tool from the Los Alamos National Laboratory (LANL) database (https://www.hiv.lanl.gov/content/sequence/HIV/mainpage.html). Sequences were uploaded to the REGA HIV-1 subtyping tool v3.46 [28] and COMET subtyping tool [29] independently for subtype classification. Maximum-likelihood phylogenetic trees were constructed using PhyML [38] to determine evolutionary relationships between patient sequences and subtype reference strains obtained from the Los Alamos HIV sequence database https://www.hiv.lanl.gov/content/sequence/GENE_CUTTER/cutter.html. In instances where the subtyping methods were not in agreement, the phylogenetic tree was used for the final subtype assignment. Figtree v1.4.4 was used to infer the branching topology.

### HIV-1 subtype recombination analysis

Inter-subtype recombination breakpoints were identified using the Jumping Profile Hidden Markov Model (jpHMM) online tool [45] and the Simplot [46] program to predict inter-subtype breakpoint coordinates. Default settings were used for jpHMM, while Simplot was optimized to a window size of 350, base pair step size of 30 and a consensus value of 50%. Each region between subtype breakpoints was extracted, trimmed, and realigned together with subtype reference sequences. Phylogenetic trees were then constructed to verify the subtype for each sequence fragment between predicted breakpoints.

### Preparation of patient-derived Gag-protease NL4-3 virus stocks

The HIV-1 patient-derived *gag-protease* amplicons from the second round nested PCR were then co-transfected with linearized NL4-3Δ*gag-protease* [47] by electroporation into CEM–GXR cells to generate chimeric viruses by homologous recombination as described previously [35]. The electroporated CEM–GXR cells maintained in R10 medium were incubated at 37 °C and 5% CO_2_ for 12 days. The percentage of virus-infected cells was monitored by flow cytometry using a FACSCalibur (BD Biosciences, New Jersey, USA) since CEM-GXR cells express green fluorescent protein (GFP) when HIV-infected [48]. Upon reaching a threshold infection rate of 25–30%, the cells were pelleted, and virus-containing supernatant was collected, aliquoted and stored at − 80 °C for subsequent cell culture experiments. To validate the viral stocks, a random subset was amplified and sequenced to confirm that the virus-derived sequences matched the original plasma HIV RNA sequences.

### Titration of virus stocks

As previously described [35], 1 × 10^6^ CEM–GXR cells were infected with 0.4 ml of virus stock and incubated for 48 h. The percentage of infected cells was then measured by flow cytometry, followed by analysis using Flowjo software (BD Biosciences, New Jersey, USA), to determine GFP expression (i.e., the percentage of infected cells) at 48 h post-infection. This percentage infected cells were used to calculate the viral volume required to achieve 0.3% (i.e., multiplicity of infection [MOI] of 0.003) infected cells after 48 h.

### Replication capacity assay

The viral replication assay was performed in duplicate as previously described [35]. Briefly, CEM-GXR cells were infected, using the same method as described in the titration assay, with the volume of virus stock calculated by the titration assay made up to a final volume of 0.4 ml using R10 medium. On days 2–6 following infection, the percentage infected cells were quantified by flow cytometry. A negative (R10 media alone) and positive (wild-type NL4-3 virus) control was included in each assay. The VRC of the chimeric viruses determined by *gag-protease* function was calculated by the mean slope of exponential growth from 3 to 6 days post infection using the semi-log method in Excel. These values were then normalized to those of the wild-type NL4-3 positive control.

### Data analysis

VRCs of patient-derived chimeric viruses were compared based on HIV-1 subtype classification and geographical region using either the Student's t-test or Mann–Whitney *U* test if two groups were compared, or ANOVA with Tukey post-hoc tests where more than two groups were compared. ANOVA was used to test for differences in VRC across each group of the major HLA class I genes (A, B and C) for participants infected with subtype A1 from East Africa, while the Student's t-test was used to compare differences in VRC values between participants that expressed specific class I HLA alleles and those that did not express such alleles. HLA class I alleles expressed in a minimum of 5 individuals were included. These analyses were performed using GraphPad Prism v.8.4.3 (GraphPad Software, San Diego, California, USA). A multivariable linear regression model performed using Stata 15 (StataCorp LLC, Texas, USA), was used to assess the relationship between subtype and VRC after adjusting for potential confounders. A variable was included in the final model if its inclusion resulted in a 10% or greater change in the coefficients of the subtype category variable. Multiple imputation using chained equations, was used to assess the sensitivity of results to missing data. Codon-by-codon Mann–Whitney *U* tests with *q* values (available at https://bblab-hivresearchtools.ca/django/tools/codon_by_codon/) were used to identify specific amino acid variants associated with increased or decreased *gag-protease*-driven VRC.

## Results

### Study participants

Samples collected from HIV-1 chronically infected participants from West and East Africa were analyzed in this study. Of the 296 samples collected from West Africa, 213 (72%) successfully yielded *gag-protease* amplicons while 160 (66%) of the 241 samples from East Africa yielded PCR products. A total of 178 (84%) and 114 (71%) of amplified samples successfully yielded chimeric viruses and were assayed for VRC for West and East Africa, respectively. Table 1 shows a summary of the study participants demographic and clinical characteristics.

**Table 1:** Demographic and clinical characteristics of study participants

	West Africa	East Africa	*p*-value
No. of participants (% male)	213 (34%)	160 (64%)	< 0.0001^a^
Age, years (IQR)^b^	32 (27–40)	28 (24–34)	0.0006^c^
CD4 + cells, cells/mm^3^ (IQR)^b^	478 (305–619)^d^	487 (347–625)	0.274^c^
Viral load, log_10_ copies/ml (IQR)^b^	4.86 (4.35–5.37)^e^	4.44 (3.95–4.93)	< 0.0001^c^

^a^*p*-values calculated using Fisher’s exact test; ^b^medians with inter-quartile ranges shown in parentheses; ^c^*p*-values calculated using the Mann–Whitney test; ^d^only 66% of participants had CD4 + T cell count data; ^e^only 49% of participants had viral load data

### HIV-1 subtype diversity and distribution

**Fig. 1:**
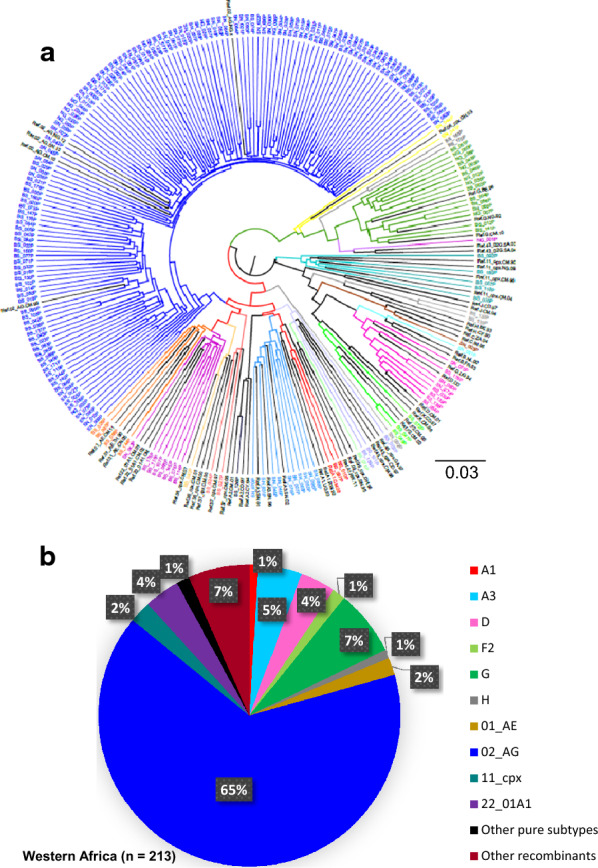
Phylogenetic analysis of *gag-protease* sequences from West Africa. Maximum-likelihood phylogenetic trees of *gag-protease* sequences **a** and subtype distribution **b** from West Africa. Phylogenetic trees were constructed using the PhyML tool on the LANL HIV database. Subtypes and inter-subtype recombinants were confirmed using reference sequences downloaded from the LANL HIV database. Branches representing subtype reference sequences are labeled in black

**Fig. 2:**
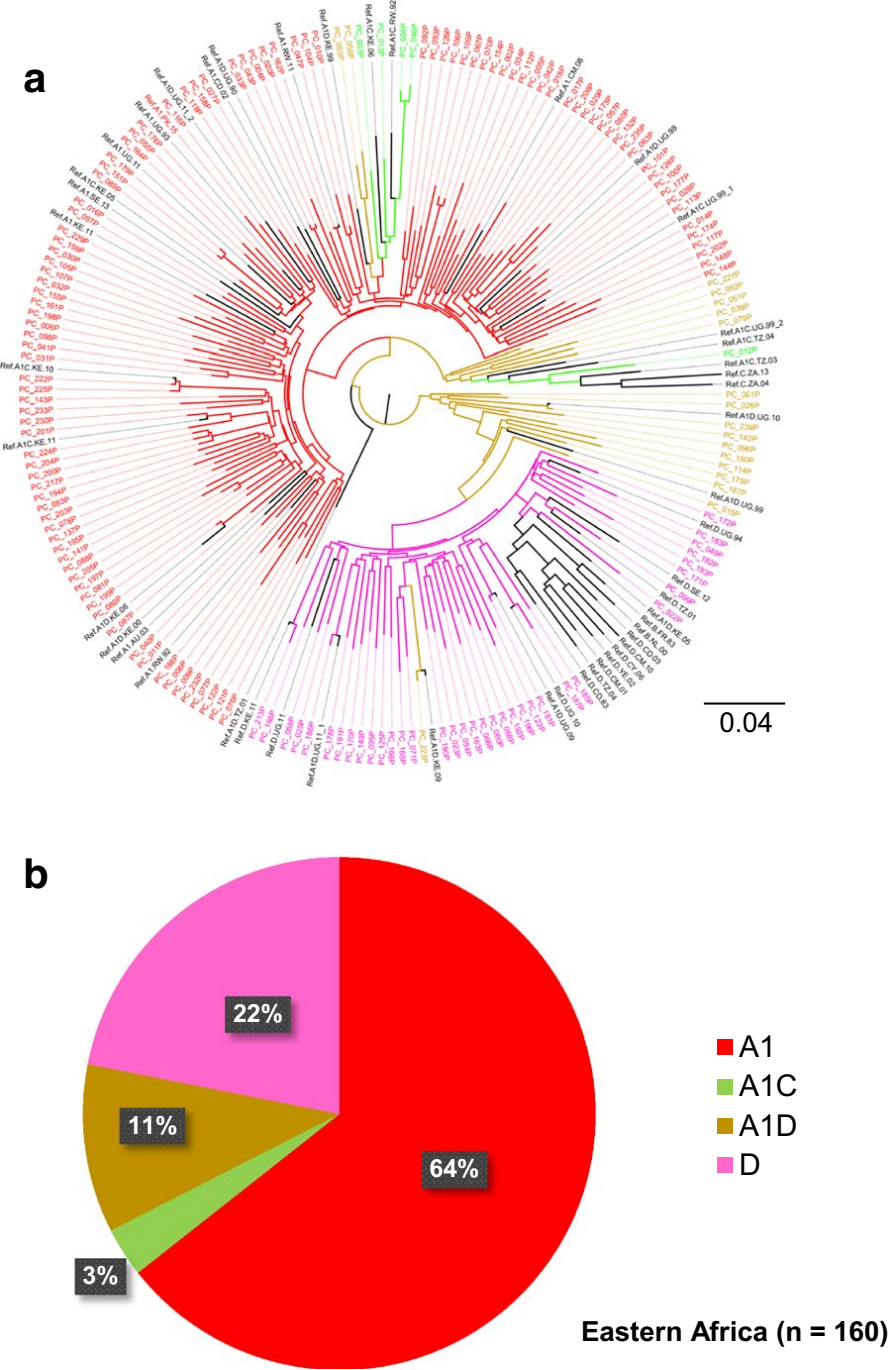
Phylogenetic analysis of *gag-protease* sequences from East Africa. Maximum-likelihood phylogenetic trees of *gag-protease* sequences **a** and subtype distribution **b** from East Africa. Phylogenetic trees were constructed using the PhyML tool on the LANL HIV database. Subtypes and inter-subtype recombinants were confirmed using reference sequences downloaded from the LANL HIV database. Branches representing subtype reference sequences are labeled in black. Samples from East Africa had been preselected for A1, D and AD based on previous *pol* sequencing [43]

A high level of subtype diversity was observed in West Africa, consistent with previous reports [6, 49]. CRF02_AG remained the most common circulating subtype in the West African isolates at 65%, followed by subtypes G and A3 at 7 and 5% respectively, while the remaining pure subtypes and CRFs were present at < 5% prevalence (Fig. 1a, b). Subtype assignment is shown in (Additional file 1: Tables S1 and S2). To facilitate comparison with West African samples and extend our previous analysis of *gag-protease* subtype in East Africa [38], we preselected subtypes A, D and AD recombinants based on previous *pol* subtyping data [43] since subtypes A, D and their recombinants have previously been demonstrated to dominate the epidemic in this region. The *gag-protease* subtyping data for the East African samples is shown in Fig. 2a, b. Though subtypes A1 and D were identified in both regions, both subtypes were significantly more prevalent in East Africa. Phylogenetic analysis showed clustering of sequences by both subtype and region (Additional file 1: Figure S1), indicating region-specific viral evolution and adaptation within subtypes.

### Gag-protease inter-subtype recombination

**Fig. 3:**
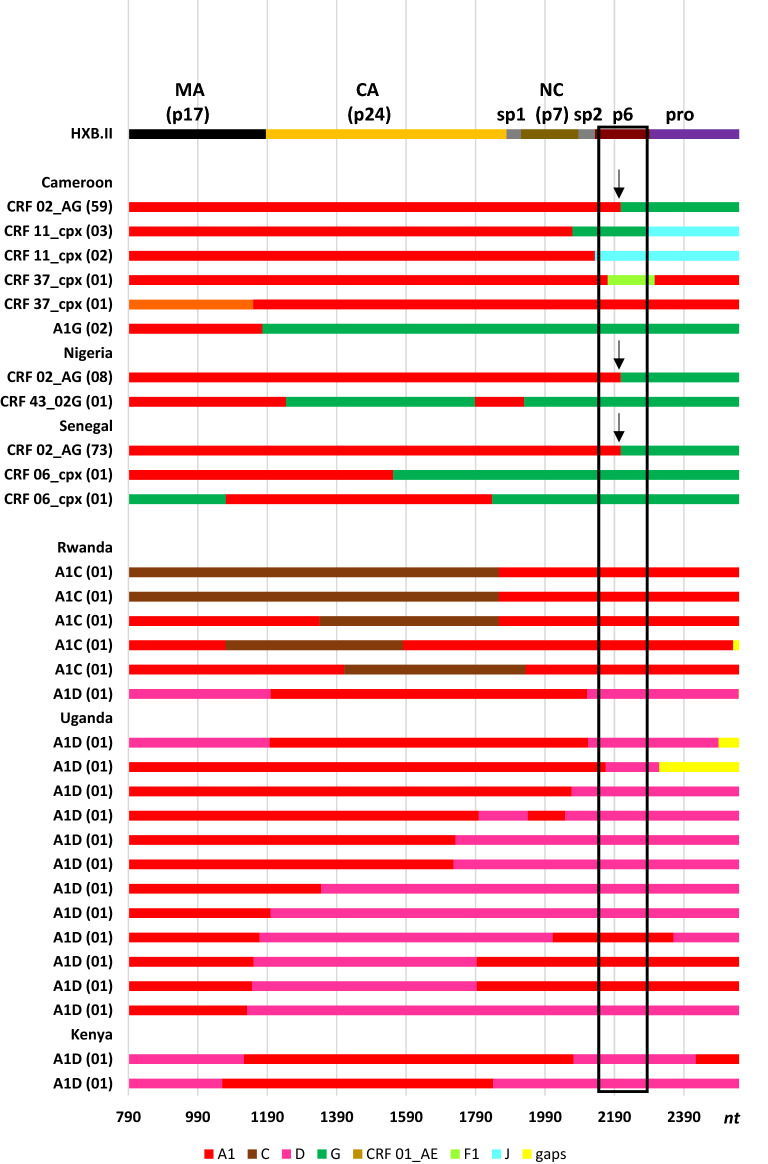
Graphic summary of inter-subtype recombination patterns within HIV-1 *gag-protease* from West and East Africa. Consensus sequence patterns of recombinants circulating within different countries in West Africa. The number of sequences identified showing a particular recombination pattern are indicated in brackets on the Y- axis, while the nucleotide position is shown on the X- axis. Missing sequence data is shown in yellow. Gene lengths and breakpoints were drawn according to the HXB2 numbering system. Black arrows show the most prevalent inter-subtype breakpoint in West Africa

Inter-subtype recombination analysis within the *gag-protease* region indicated that all HIV-1 CRFs and other recombinant forms in West and East Africa were recombinants of A1 (Fig. 3). In West Africa, the most prevalent inter-subtype recombinants were those of subtypes A1 and G, and less common inter-subtype recombinants were combinations of A1 and J, or A1, G and J mosaics, with CRF02_AG being the most common followed by CRF11_cpx. In the *gag* region, the remaining West African CRFs were close genetic relatives of subtype A1 (CRF01_AE, CRF09_cpx, CRF22_01A1, CRF36_cpx, CRF37_cpx, CRF45_cpx) or A1, G (CRF06_cpx and CRF43_02G) recombinants, with 96% (146/152) of these inter-subtype recombinants having a breakpoint within the p6 region in *gag*. In East Africa, we identified inter-subtype recombination between subtype A1 and C or A1 and D. All 5 A1C recombinants identified were from Rwanda and had a subtype A component towards the 3′ end of the sequences, while 80% (n = 12) of all A1D sequences from Uganda had a subtype D 3′ end (which included the entire p6 region). This recombination pattern is consistent with previous work from our group [38] indicating an evolutionary preference in A1D inter-subtype recombinant Gag sequences for subtype D towards the 3′ end of the sequence.

### *Gag-protease* driven replication capacity of West and East African subtypes

**Fig. 4:**
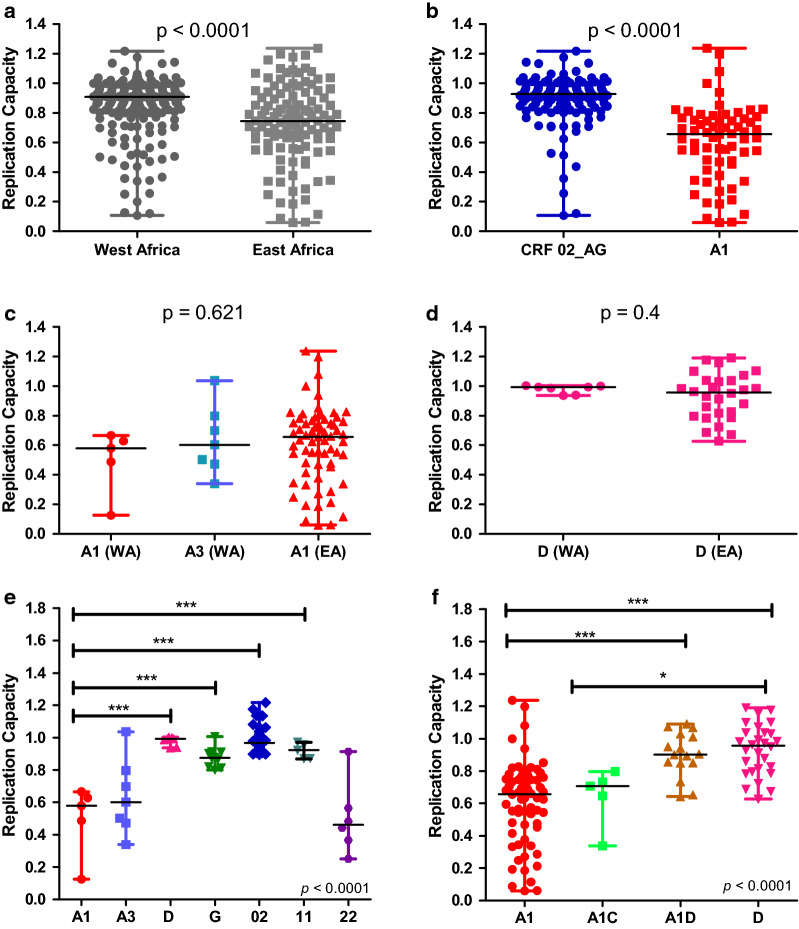
Comparison of Gag-protease-driven VRC by geographic region and HIV-1 subtype.** a** Prevalent chronically infected patient-derived *gag-protease* chimeric viruses from West Africa have significantly higher VRC compared to those from East Africa. **b** The predominant subtype in West Africa (CRF02_AG) displayed higher VRC than the predominant subtype (A1) in East Africa. **c**, **d** There was no significant difference in VRC between subtype A viruses from West and East Africa, and neither between subtype D viruses from West and East Africa. **e**, **f** Significant differences in VRC were observed between subtypes in both West Africa and East Africa. In panel E, the significant differences between A1 and the other subtypes were also observed between A3/CRF22_01A1 and the other subtypes used in the analysis. In all panels, the Mann Whitney *U* test was used for two group comparisons, and ANOVA was used where more than two groups were compared. The Mann–Whitney and ANOVA p values are shown, while the Tukey multiple comparison test p values are indicated with asterisks, where *p < 0.05, **p < 0.001 and ***p < 0.0001. CRFs are represented by their respective numbers (CRF02_AG, CRF11_cpx, CRF22_01A1)

Overall, prevalent viral isolates from West Africa had significantly higher VRC compared to those from East Africa (Fig. 4a). A subsequent comparison of the most prevalent circulating subtype in each region showed that CRF02_AG, which was most predominant in West Africa had a higher VRC than subtype A1 which was the most predominant subtype in the East African samples (Fig. 4b), indicating that the prevalent subtypes were driving the overall difference in VRC observed between both regions. Consistent with this idea, intra-subtype comparison of A1 and D between regions showed no difference in VRC according to region (Fig. 4c, d). In West Africa, pure subtype A1 and its close genetic relative A3/CRF22_01A1 had the lowest *gag-protease* driven VRC when compared to subtypes D, G, CRF02_AG, and CRF11_cpx (Fig. 4e). In East Africa, pure subtype A1 also had the lowest *gag-protease* driven VRC when compared to other subtypes, and a hierarchy of A/A1C < A1D < D was observed (Fig. 4f), which is consistent with previous reports by our group [38].

### Multivariate regression analysis (West and East Africa)

Men have been shown to have higher viral loads than women [23, 50]. Viral load as well as CD4 + T cell count are significantly associated with *gag-protease* driven VRC [35, 37] Therefore, a multivariable linear regression was used to assess the relationship between subtype and VRC after adjusting for potential confounders, including participant’s sex, age, viral load and CD4 + T cell counts (Table 1).

In West Africa, the most frequently observed subtype was CRF02_AG which was observed in 139 (65%) of participants. In the univariate analysis, subtypes A1, A1G recombinants, A3 and CRF22_01A1 were all associated with significantly lower VRC when compared to CRF 02_AG. Less than 25% of participants had complete information on all the following parameters: CD4 + count, viral load, and VRC, rendering the construction of a multivariable or imputation model challenging. In view of country-specific missing data patterns, we constructed separate multivariable models for Cameroon and Senegal (but not Nigeria due to the small sample size) to gain insight into the effect of subtype on VRC while adjusting for confounders (Table 2). The country-specific multivariate models collectively supported the significant findings in the univariate analysis: A1 (0.49, *p* < 0.0001), A1G recombinants (0.35, *p* = 0.01), A3 (0.2, *p* = 0.03) and CRF22_01A1 (0.4, *p* < 0.0001) had significantly lower mean VRC compared to CRF02_AG.

**Table 2:** Linear regression models investigating the relationship between HIV-1 subtype and VRC for West Africa

	Univariate model		Multivariate model			
	West Africa		Cameroon		Senegal	
Observations	166		77		66	
Prob > f	< 0.0001		< 0.0001		0.022	
r^2^	0.290		0.653		0.193	
^a^Subtypes	Co-efficient	*p*-value	Co-efficient	*p*-value	Co-efficient	*p*-value
A1	− 0.388	< 0.0001	− 0.489	< 0.0001	–	–
A1G^b^	− 0.190	0.013	− 0.012	0.854	− 0.352	0.010
A3	− 0.250	< 0.0001	–	–	− 0.199	0.032
CRF 11_cpx	0.033	0.691	− 0.006	0.907	–	–
CRF 22_01A1	− 0.038	< 0.0001	− 0.398	< 0.0001	–	–
D	0.093	0.187	0.024	0.681	0.137	0.308
G	− 0.017	0.775	− 0.098	0.223	− 0.067	0.768
Meta data						
Age	–	–	− 0.001	0.629	–	–
Log VL	–	–	0.033	0.054	–	–
CD4 + count	–	–	–	–	0.000	0.137

^a^CRF02_AG is the reference subtype. ^b^A1G is not a CRF

In East Africa, a total of 114 participants had complete information on VRC and were included in the regression models. The A1D and D subtypes were associated with significantly higher VRC values, after adjusting for age, sex, country, CD4 + count and viral load. Using multiple imputation with multivariate regression the dataset was imputed 50 times and estimates were combined on the imputed dataset, yielding revised regression estimates. After multiple imputation A1D had a 0.24 significantly higher mean VRC compared to A1 (*p* < 0.0001), while subtype D had a 0.29 significantly higher mean VRC compared to A1 (*p* < 0.0001) (Table 3) after adjusting for age, sex, country, CD4 + T cell count and viral load.

**Table 3:** Linear regression models investigating the relationship between HIV-1 subtype and VRC for East Africa

	Univariate model		Multivariate model	
Observations	114		111	
Prob > f	< 0.0001		< 0.0001	
r^2^	0.30		0.34	
^a^Subtypes	Co-efficient	*p*-value	Co-efficient	*p*-value
A1C	0.07	0.5	0.113	0.298
A1D	0.25	< 0.0001	0.239	< 0.0001
D	0.31	< 0.0001	0.292	< 0.0001
^b^Country				
Rwanda	–	–	− 0.075	0.272
Uganda	–	–	− 0.032	0.622
Meta data				
Age	–	–	− 0.003	0.175
Log VL	–	–	0.012	0.727
CD4 + count	–	–	< 0.0001	0.858
Sex	–	–	− 0.034	0.481

^a^A1 is the reference subtype. ^b^Kenya is the reference country

### Association of HLA class I alleles with *gag-protease*-driven replication capacity

**Fig. 5:**
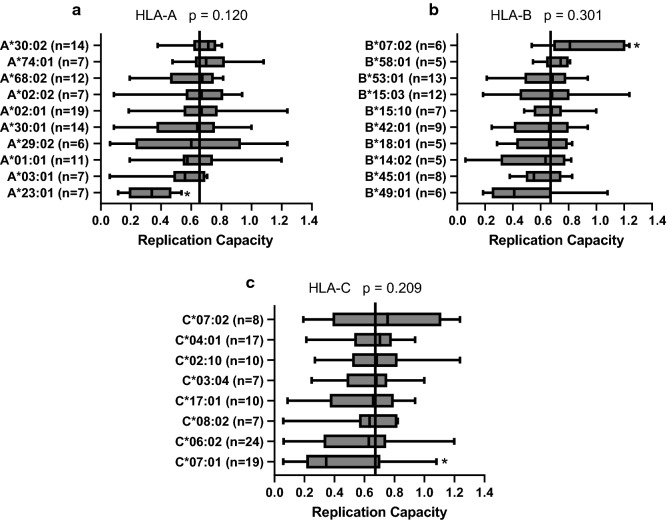
Associations between HLA class I allele expression and *Gag-protease* driven VRC. *Gag-protease* driven VRC of chronically infected participants from East Africa with subtype A1 infection were grouped according to **a** HLA-A, **b** HLA-B and **c** HLA-C class I alleles expressed. The box plots display VRC results arranged from lowest median VRC at the bottom and the highest median VRC at the top. Boundaries of the boxes indicate the interquartile ranges, while the whiskers display the maximum and minimum VRC values. The continuous vertical line on each graph indicates the median VRC (0.6560) for all subtype A1 viruses from East Africa. HLA-I alleles with a minimum of n = 5 are shown. Asterisks indicate HLA alleles that are significantly (*p* < 0.05) associated with either higher or lower VRC (Student's *t*-test). Median value for subtype A1 VRC is represented by the vertical line shown at the middle of the graph

Genome wide association studies have demonstrated that the human leukocyte antigen class I (HLA-I) alleles are the most significant genetic determinant of clinical outcome in HIV-1 infection [51]. To investigate the impact of HLA-I allele expression on the Gag-protease driven VRC of subtype A1 patient-derived isolates from East Africa, VRC data were grouped according to HLA-I alleles expressed by the hosts for each of the class I loci (Fig. 5). VRC did not differ significantly across alleles in any of the class I loci, however analysis of individual HLA-I alleles showed that HLA-A*23:01 and HLA-C*07:01 were associated with significantly lower VRC (Student's *t*-test, *p* = 0.0014 and *p* = 0.002) respectively, while HLA-B*07:02 was associated with significantly higher VRC (Student's *t*-test, *p* = 0.004).

### Gag p6 subtype predicts VRC

**Fig. 6:**
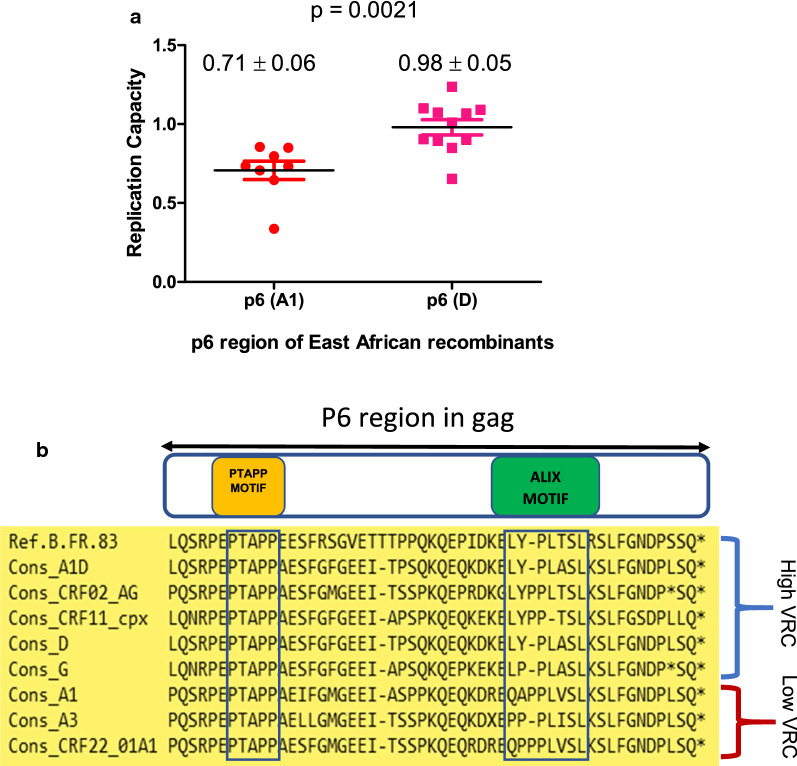
Analysis of the amino acid sequence of the p6 region of Gag. **a** Comparative analysis of VRC of East African recombinants with A1 and D components respectively within the p6 region of *gag* showed a significant difference (*p* < 0.05) between both groups. **b** Consensus amino acid sequence of subtypes A1, A3, CRF02_AG, CRF11_cpx, CRF22_01A1, D, and G show mutations within the Alix motif showing which subtype sequences corresponded to high or low VRC. VRC is defined as high or low if it is greater or less than the median VRC value (0.86) of the total population studied irrespective of region

Recombination patterns in West and East African sequences showed a preference for subtypes D, G or J rather than the predominant subtype A in the p6 region of Gag, highlighting an important evolutionary trait that may impact VRC. In support of this, a multiple sequence alignment of URFs from East Africa was performed, and the p6 region of the alignment was extracted and confirmed by phylogenetic analysis. Corresponding VRC data were categorized based on subtype of the p6 region. The results showed that in East Africa (Fig. 6a), p6 regions with subtype A1 had significantly lower (p = 0.0021) VRC than those with subtype D. A corresponding analysis could not be done for West African sequences as we lacked sufficient sequences with a subtype A1 p6 region for the West African CRFs.

To investigate subtype-specific amino acid sequences in the p6 region of *Gag*, consensus sequences of individual subtypes irrespective of region were generated A1 (n = 104), A1D (n = 16), A3 (n = 10), CRF02_AG (n = 138), CRF11_cpx (n = 5), CRF22_01A1 (n = 8), D (n = 43) and G (n = 15) and aligned with the HXB2 subtype B sequence as a reference. While amino acid sequence in the PTAP domain remained conserved irrespective of subtype, there were marked subtype-specific variations in the LYPLASL domain in the Alix budding motif. The subtypes with above median VRC are conserved for the LY residues except for subtype G which has LP as the consensus at this position in our sequences, while the subtypes with below median VRC have other amino acids at the same position (Fig. 6b), suggesting, along with previous reports [38, 52], that variation in the Alix motif could impact VRC.

### Codon-by-codon analysis

An exploratory codon-by-codon analysis was performed to identify amino acid polymorphic variants associated with differences in VRC for the predominant subtypes in West (CRF02_AG) and East (A1) Africa using methods previously described [53]. The analysis identified several amino acid polymorphisms that associated with altered VRC at *p* < 0.05, although after correcting for multiple codon comparisons none of the associations met the significant threshold of *q* < 0.2 (Tables 4 and 5).

**Table 4:** Codon-by-codon analysis of Gag-protease polymorphisms and viral replication capacity (VRC) for CRF 02_AG

^a^Consensus AA/Codon	^b^AA Signal	Median VRC with AA	Median VRC without AA	No. of patients with AA	No. of patients without AA	*p*-value	*q*-value	A-list epitope
L34	L	0.91	0.95	83	27	0.022	0.525	KW9 (HLA A*24:02), HL9, LF11 (HLA A*30)
L34	I	0.95	0.91	27	83	0.022	0.525	
A83	A	0.93	0.73	109	6	0.015	0.525	RY11 (HLA B*08:01), SL9 (HLA A*02:01, HLA A*02:02, HLA A*02:05), SY10 (HLA A*02:01), LY9 (HLA A*29:02, HLA B*44:03)
A83	V	0.73	0.93	6	109	0.015	0.525	
S125	S	0.91	0.95	69	40	0.029	0.525	NY9 (HLA B*35:01)
A146	N	1.00	0.91	13	101	0.006	0.514	GI8 (HLA B*13:02), HL9 (HLA B*15:10), QW11 (HLA A*25:01), IW9 (HLA B*57:01, HLA B*63)
E260	D	0.97	0.91	19	96	0.011	0.525	NY10, PY9 (HLA B*35:01),EI8 (HLA B*08:01)
E260	E	0.91	0.97	94	21	0.017	0.525	
V267	V	0.91	0.94	58	56	0.032	0.525	EI10(HLA B*08:01), RK10 (HLA B*27:03), KK10 (HLA B*27:05)
V267	I	0.94	0.91	56	58	0.032	0.525	
S332	A	1.04	0.92	5	110	0.005	0.514	DL9 (HLA B*08:01)
R335	R	0.91	0.95	73	40	0.025	0.525	DL9 (HLA B*08:01)
R335	K	0.95	0.91	40	73	0.025	0.525	

^a^This column represents the consensus amino acid at specified at that locus; ^b^This column represents the amino acid variant being reported by the codon analysis; ^c^A-list epitope as available from http://www.lanl.com

**Table 5:** Codon-by-codon analysis of Gag-protease polymorphisms and viral replication capacity (VRC) for A1

Consensus AA/Codon	AA Signal	Median VRC with AA	Median VRC without AA	No. of patients with AA	No. of patients without AA	*p*-value	*q*-value	A-list epitope
K12	K	0.68	0.23	52	8	0.045	0.862	GI9 (HLA B*40:02)
K28	K	0.69	0.47	45	13	0.008	0.862	RK9 and RY10 (HLA A*03:01)
A67	A	0.63	0.82	57	5	0.023	0.862	–
K69	Q	0.77	0.63	8	52	0.048	0.862	–
S125	N	0.76	0.63	8	52	0.042	0.862	NY9 (HLA B*35:01)
L147	I	0.79	0.63	5	55	0.039	0.862	GI8 (HLA B*13:02), HL9 (HLA B*15:10), QW11 (HLA A*25:01), IW9 (HLA B*57:01, B*63)
V158	V	0.63	0.75	54	9	0.038	0.862	VF (HLA B*15:03)
V158	A	0.75	0.63	9	54	0.038	0.862	
I223	I	0.63	0.73	49	14	0.028	0.862	HA9 (HLA B*35:01, B7)
R380	K	0.75	0.63	9	53	0.036	0.862	–
P497	L	0.54	0.68	16	42	0.021	0.862	–

^a^This column represents the consensus amino acid at specified at that locus; ^b^This column represents the amino acid variant being reported by the codon analysis; ^c^A-list epitope as available from http://www.lanl.com

In CRF02_AG sequences, A83V, a previously inferred escape variant [54] in known CTL epitopes in the p17 region of *gag* was the only non-consensus amino acid variant associated with a lower VRC (Table 4). This result was consistent with previous reports linking A83V to reduced viral fitness in subtypes B and CRF01_AE [54, 55]. In subtype A sequences, the only non-consensus polymorphism associated with decreased VRC was P497L, while non-consensus amino acid variants K69Q, S125N, L147I, A158V and R380K, were associated with significantly higher VRC (Table 5). However, the consensus amino acids at positions 12 and 28 were associated with increased VRC, which suggests that mutations at these codons, within known CTL epitopes, confer a fitness cost. Consistent with this, polymorphisms at codon 28 have been reported to alter VRC in other subtypes [40, 56]. Further work is required to validate these findings since the relatively small number of sequences available for analysis resulted in limited statistical power.

## Discussion

A hallmark of the HIV-1 epidemic in sub-Saharan Africa is the region-specific differences in prevalence such that southern African countries tend to have the highest prevalence rates followed by East African countries, with West and Central African countries having the lowest prevalence rates [57–59]. It is also noteworthy that HIV-1 subtypes are unevenly distributed across the continent, with subtype C predominant in southern Africa, subtypes A, D and C common in East Africa and almost all subtypes present in West and Central Africa [59, 60]. The possible contribution of viral genetic and functional characteristics to the uneven distribution of subtypes is unresolved but differences in transmission efficiency and rate of disease progression have been confirmed in epidemiological and clinical studies, suggesting that viral factors may partially explain the heterogeneity in prevalence and uneven spread of HIV-1 subtypes within the continent [18, 19, 26, 38, 61–63]. Here, using samples from HIV-1 chronically infected individuals from 3 West African countries, we confirm findings from previous studies that the epidemic in this region is genetically heterogenous with CRF02_AG (65%) the predominant circulating strain but with other subtypes and recombinant forms each contributing a significant proportion of the epidemic in the region [7, 9, 59]. Our study was not designed to explore the genetic diversity of the HIV-1 epidemic in East Africa since the samples analyzed for this study were preselected to be either subtypes A, D or their recombinants based on previous *pol* sequencing [43], however, numerous other studies have demonstrated a far less diverse epidemic in East Africa dominated by subtypes A, D, C and their recombinant forms [19, 20, 38, 64, 65].

Recombination analyses of the West African isolates identified several CRFs, consistent with the multiple pure subtypes that have been identified within the region [66]. In West Africa, most recombinants comprised of A1 and G genetic fragments and breakpoint analysis suggested that recombination patterns were not random, with a strong preference for subtype G or J over A1 at the 3′-end of most recombinant sequences, with a recombination hotspot around the Gag p6 region. A similar trend was noted for the East African isolates where there was also a preference for subtype D over A for the Gag p6 region in recombinant sequences. Overall, the data suggested that subtype A1 in both West and East Africa has a high propensity for inter-subtype recombination with non-A subtypes at the 3′ end, preferred particularly from around the Gag p6 region. We hypothesize that this could be a mechanism to facilitate the adaptation of HIV-1 subtype A into a fitter virus. Indeed, it has been reported that throughout sub-Saharan Africa, pure forms of subtype A are in decline, with a concomitant increase in its recombinants [59].

The notion that recombination may be a non-random mechanism to enhance viral fitness is backed up by our VRC data. In West and East Africa, *gag-protease*-driven VRC differed by HIV-1 subtype with a clear hierarchy where A1/A3 had generally lower VRC compared to pure subtypes D and G or A1 recombinants (although this was not the case for A1/C recombinants and CRF22_01A1). Our data is thus consistent with previous studies of Gag-protease functional differences among HIV-1 subtypes and recombinants that may also extend to other HIV-1 proteins [33, 35, 53, 67, 68]. Enhanced *gag-protease*-driven VRC has been associated with faster rate of CD4 + T cell decline and disease progression [69], and our data is consistent with previously reported subtype-specific differences in disease progression [17–19, 22].

Interestingly, in East Africa, it is subtype A1, with a lower replication capacity than subtype D that has been reported to be more transmissible; despite the latter being associated with faster disease progression [20, 21, 38, 64, 65]. This observation, together with reports that the HIV-1 transmission bottleneck may favor consensus-like viruses, which have been associated with lower replication capacity [35, 37, 39, 40], led us to our hypothesis that West African viral isolates, where HIV prevalence is low may have higher replication capacity compared to the viruses from East Africa where prevalence is higher. Although we found that West African viruses have higher overall viral replication capacity compared to East African viruses, these differences are explained by the subtypes prevalent in these regions and are not region-specific per se. Moreover, in contrast to the situation in East Africa where a virus strain (subtype A) with lower replication capacity predominates, CRF02_AG is the predominant strain in West Africa and yet it has a higher viral replication capacity than other strains prevalent in the region. Overall, our data suggest that viral replication capacity alone does not explain the epidemiological success of a virus strain. Our data highlights the need for further studies to better understand the viral determinants that may underlie transmissibility and virulence.

Recombination patterns suggested that the 3′ region of the Gag protein is a recombination hotspot particularly for A1 recombinants. Specifically, the non-A subtype was preferred for the p6 region of Gag. The p6 contains two well studied segments, the PTAP and LYPLASL domains which interact with the host cell TSG101 and Alix factors respectively to facilitate efficient virion maturation and budding from host cells [52, 70]. Whereas the PTAP motif showed no amino acid variation across subtypes, the LYPLASL motif showed variation across subtypes which corresponded to either low or high VRC and a comparative analysis of East African recombinants with A1 and D components within the p6 region showed a lower VRC for those with subtype A1 (*p* < 0.05). We and others have shown previously that variation in the Alix budding motif in Gag affects VRC [38, 71]. Further work to interrogate the effect of these subtype-specific amino acid variations within the LYPLASL motifs on VRC is warranted.

Finally, our study identified HLA-A class I alleles that have differential impact on HIV-1 VRC for HIV-1 subtype A, the predominant subtype in East Africa which suggest the need for further studies to identify regions of viral vulnerability for HIV-1 vaccine design strategies. Furthermore, viral replication capacities did not differ across HLA class I loci for HIV-1 subtype A in East Africa, even for HLA-B alleles, contrary to earlier observations, particularly in subtype B and C infections, of strong selective pressure by certain HLA-B alleles that may impact replication capacity [13, 35, 56, 72]. However, individually, HLA-A*23:01 and HLA-C*07:01 were associated with lower replication capacity, suggesting an underappreciated role in immune selection pressure on HIV-1 subtype A by certain HLA-A and HLA-C alleles. This finding appears consistent with previous findings of subtype-specific differences in HLA-driven viral evolution that may have consequences for natural and vaccine-mediated immunity [73]. Overall, the data highlight the need for further studies to identify mechanisms of immune control or regions of viral vulnerability by HLA alleles common in regions of the world with non-B and C subtypes as this knowledge may be useful for universal virus attenuation-based vaccine strategies.

Limitations of the current study include missing clinical data such as CD4 + T cell counts and HLA-I genotypes, for the West African samples, thus limiting the extent of statistical analysis. East African samples were preselected for subtypes A, D and their recombinants based on prior *pol* genotyping, therefore not allowing comprehensive inter-subtype comparison of the strains that constitute the East Africa epidemic. It should also be noted that all replication assays were performed in the subtype B pNL4-3∆*gag-protease* backbone, however, we have previously demonstrated that VRC data generated from this assay is generally reflective of whole virus isolates in HIV-1 subtype C [35, 38]. A higher sample size would also have benefited the robustness of the codon-by-codon analysis to identify amino acids associated with altered VRC for different subtypes and recombinants.

## Conclusions

Overall, in this study we show that the most prevalent HIV-1 viruses from West Africa display higher replication capacities than those from East Africa, consistent with the hypothesis that higher prevalence is associated with lower replication capacity of circulating strains. Subtype-specific differences in replication capacity in agreement with previous studies were noted, and consistent with reported differences in the rate of clinical disease progression. Our study identified inter-subtype recombination patterns as a driver of virus replication capacity differences and identified HLA class I alleles and specific amino acids that may alter virus replication, information that may be relevant for HIV-1 vaccine design strategies.

## Supplementary Information

**Additional file 1.** Additional tables and figure.

## Data Availability

The datasets used and analysed during this study are available from the corresponding author.

## Abbreviations

VRC: Viral replication capacity
CRF: Circulating recombinant Form
URF: Unique recombinant Form
jpHMM: Jumping profile Hidden Markov model
GFP: Green fluorescent protein
HLA: Human leucocyte antigen
cDNA: Complementary deoxyribonucleic acid
RT-PCR: Reverse transcriptase polymerase chain reaction
LANL: Los Alamos National Laboratory
MOI: Multiplicity of infection
HIV-1: Human immunodeficiency virus type 1
