# Supplementary material for: Subtype-specific differences in Gag-protease replication capacity of HIV-1 isolates from East and West Africa
Source: Retrovirology. 2021 May 5;18:11. doi: 10.1186/s12977-021-00554-4 (PMC8097975; doi:10.1186/s12977-021-00554-4)
Supplement: Supplementary file 1 — Additional file 1. Additional tables and figure. [file 12977_2021_554_MOESM1_ESM.docx]

**Supplementary Table 1: West African samples *gag–protease* subtype assignment**

| SAMPLE ID | REGA | COMET | Mphy |  |
| --- | --- | --- | --- | --- |
|  |  |  |  |  |
| BS 001 | A (02_AG) | A1 (check for 02_AG)* | CRF 02_AG |  |
| BS 002 | A (A1) | A1 | A1-like |  |
| BS 003 | G | G | G |  |
| BS 004 | G | G | G |  |
| BS 005 | A (02_AG) | 02_AG | CRF 02_AG |  |
| BS 006 | A (02_AG) | A1 (check for 02_AG)* | CRF 02_AG |  |
| BS 009 | A (02_AG) | A1 (check for 02_AG)* | CRF 02_AG |  |
| BS 010 | A (A1) | A1 | A1 |  |
| BS 011 | CRF 02_AG | ? | CRF 02_AG |  |
| BS 012 | G | G | G |  |
| BS 013 | A (A1) | 02_AG | CRF 02_AG |  |
| BS 014 | A (02_AG) | 02_AG | CRF 02_AG |  |
| BS 016 | A (02_AG) | 02_AG | CRF 02_AG |  |
| BS 019 | A (02_AG) | 02_AG | CRF 02_AG |  |
| BS 020 | A (02_AG) | 02_AG | CRF 02_AG |  |
| BS 021 | A (02_AG) | A1 (check for 02_AG)* | CRF 02_AG |  |
| BS 022 | A (02_AG) | 02_AG | CRF 02_AG |  |
| BS 023 | A (02_AG) | 02_AG | CRF 02_AG |  |
| BS 024 | A (37_cpx) | 37_CPX | CRF 37_CPX |  |
| BS 025 | A (02_AG) | 02_AG | CRF 02_AG |  |
| BS 026 | A (A1) | 01_AE | CRF 01_AE |  |
| BS 027 | A (37_cpx) | 02_AG | CRF 37_CPX |  |
| BS 029 | A (02_AG) | A1 (check for 02_AG)* | CRF 02_AG |  |
| BS 030 | D | D | D |  |
| BS 032 | A (02_AG) | 02_AG | CRF 02_AG |  |
| BS 035 | 11_CPX, A1 | CRF 11_CPX | CRF 11_CPX |  |
| BS 038 | A (02_AG) | 02_AG | CRF 02_AG |  |
| BS 039 | A (A1) | 02_AG | CRF 02_AG |  |
| BS 040 | A (A1) | CRF 36_CPX | CRF 36_CPX |  |
| BS 042 | A (02_AG) | A (02_AG) | CRF 02_AG |  |
| BS 043 | A (02_AG) | A1 (check for 02_AG)* | CRF 02_AG |  |
| BS 044 | A (02_AG) | 02_AG | CRF 02_AG |  |
| BS 045 | A (02_AG) | A1 (check for 02_AG)* | CRF 02_AG |  |
| BS 046 | G | G | G |  |
| BS 047 | F1,K | ? | F2 |  |
| BS 048 | G | G | G |  |
| BS 049 | F2, K | F2 | F2 |  |
| BS 050 | A (02_AG) | 02_AG | CRF 02_AG |  |
| BS 051 | G | G | G |  |
| BS 053 | A (02_AG) | A1 (check for 02_AG)* | CRF 02_AG |  |
| BS 054 | D | D | D |  |
| BS 055 | A (02_AG) | 02_AG | CRF 02_AG |  |
| BS 056 | A (02_AG) | A1 (check for 02_AG)* | CRF 02_AG |  |
| BS 057 | CRF 11_CPX-like | 11_cpx | CRF 11_CPX |  |
| BS 064 | A (02_AG) | A1 (check for 02_AG)* | CRF 02_AG |  |
| BS 065 | A (A1) | ? | CRF 22_01A1 |  |
| BS 066 | A (02_AG) | A1 (check for 02_AG)* | CRF 02_AG |  |
| BS 071 | A (02_AG) | A1 (check for 02_AG)* | CRF 02_AG |  |
| BS 072 | A1, F1, K | ? | F2 |  |
| BS 073 | A (02_AG) | A1 (check for 02_AG)* | CRF 02_AG |  |
| BS 074 | A (02_AG) | 02_AG | CRF 02_AG |  |
| BS 075 | A (02_AG) | 02_AG | CRF 02_AG |  |
| BS 076 | A (A1) | A1 (check for 09_cpx)* | CRF 09_cpx |  |
| BS 077 | A (02_AG) | A1 (check for 02_AG)* | CRF 02_AG |  |
| BS 078 | A (01_AE) | ? | CRF 22_01A1 |  |
| BS 081 | A (02_AG) | 02_AG | CRF 02_AG |  |
| BS 083 | A (02_AG) | 02_AG | CRF 02_AG |  |
| BS 085 | A (02_AG) | 02_AG | CRF 02_AG |  |
| BS 086 | A (02_AG) | 02_AG | CRF 02_AG |  |
| BS 088 | A (01_AE) | 01_AE | CRF 01_AE |  |
| BS 090 | A (A1) | 01_AE | CRF 01_AE |  |
| BS 092 | CRF 11_cpx | ? | CRF 11_CPX |  |
| BS 094 | A (02_AG) | 02_AG | CRF 02_AG |  |
| BS 096 | A (02_AG) | 02_AG | CRF 02_AG |  |
| BS 097 | A (A1) | A1 | A1 |  |
| BS 099 | A (02_AG) | 02_AG | CRF 02_AG |  |
| BS 101 | A (A1) | A1 | CRF 45_cpx |  |
| BS 102 | A (02_AG) | 02_AG | CRF 02_AG |  |
| BS 105 | A (02_AG) | 02_AG | CRF 02_AG |  |
| BS 107 | A (A1) | 01_AE | CRF 22_01A1 |  |
| BS 114 | A (A1) | 01_AE | CRF 22_01A1 |  |
| BS 115 | A (A1) | ? | CRF 22_01A1 |  |
| BS 117 | A (02_AG) | A1 (check for 02_AG)* | CRF 02_AG |  |
| BS 118 | CRF 11_CPX | 11_cpx | CRF 11_CPX |  |
| BS 119 | A (02_AG) | 02_AG | CRF 02_AG |  |
| BS 120 | CRF 02_AG | 02_AG | CRF 02_AG |  |
| BS 123 | A (02_AG) | 02_AG | CRF 02_AG |  |
| BS 125 | A (02_AG) | 02_AG | CRF 02_AG |  |
| BS 126 | A2 | A2 | A2 |  |
| BS 129 | CRF 02_AG | 02_AG | CRF 02_AG |  |
| BS 130 | CRF 02_AG | 02_AG | CRF 02_AG |  |
| BS 133 | H | H | H |  |
| BS 135 | H | H | H |  |
| BS 136 | D | D | D |  |
| BS 137 | A (02_AG) | A1 (check for 02_AG)* | CRF 02_AG |  |
| BS 139 | D | D | D |  |
| BS 140 | A (02_AG) | 02_AG | CRF 02_AG |  |
| BS 141 | G | G | G |  |
| BS 142 | A (02_AG) | 02_AG | CRF 02_AG |  |
| BS 143 | A (02_AG) | 02_AG | CRF 02_AG |  |
| BS 144 | A(01_AE) | A1 | CRF 01_AE |  |
| BS 145 | A (01_AE) | 01_AE | CRF 22_01A1 |  |
| BS 147 | A (02_AG) | 02_AG | CRF 02_AG |  |
| BS 159 | A (A1) | A1 | CRF 45_cpx |  |
| BS 160 | CRF 11_CPX | 11_cpx | CRF 11_CPX |  |
| BS 163 | A (02_AG) | A1 (check for 02_AG)* | A1, G |  |
| BS 166 | G, A1 | ? | A1, G |  |
| BS 168 | CRF 02_AG | 02_AG | CRF 02_AG |  |
| BS 170 | G, A1 | ? | A1, G |  |
| BS 175 | A (01_AE) | 01_AE | CRF 22_01A1 |  |
| BS 178 | A (02_AG) | 02_AG | CRF 02_AG |  |
| BS 179 | D | D | D |  |
| BS 180 | A (02_AG) | A1 (check for 02_AG)* | CRF 02_AG |  |
| NG 001 | G, A1* | ? | CRF 43_02G |  |
| NG_002 | CRF 02_AG-like | ? | CRF 02_AG |  |
| NG 003 | G | G | G |  |
| NG_006 | CRF 02_AG | 02_AG | CRF 02_AG |  |
| NG 007 | G | ? | G |  |
| NG 012 | G | G | G |  |
| NG 032 | CRF 02_AG | A1(check for 02_AG)* | CRF 02_AG |  |
| NG 033 | CRF 02_AG | CRF 02_AG | CRF 02_AG |  |
| NG 035 | CRF 02_AG | 02_AG | CRF 02_AG |  |
| NG 036 | G | G | G |  |
| NG 038 | G | G | G |  |
| NG 039 | CRF 02_AG | 02_AG | CRF 02_AG |  |
| NG 046 | CRF 02_AG | CRF 02_AG | CRF 02_AG |  |
| NG 050 | G | G | G |  |
| NG 053 | CRF 02_AG | CRF 02_AG | CRF 02_AG |  |
| NG 069 | 02_AG, G | ? | CRF 02_AG |  |
| SN 001 | CRF 02_AG | 02_AG | CRF 02_AG |  |
| SN 002 | CRF 02_AG | 02_AG | CRF 02_AG |  |
| SN 003 | C | C | C |  |
| SN 004 | A (02_AG) | A1/02_AG* | CRF 02_AG |  |
| SN 005 | A (02_AG) | 02_AG | CRF 02_AG |  |
| SN 006 | CRF 02_AG | 02_AG | CRF 02_AG |  |
| SN 007 | A (A1) | A1 | A3 |  |
| SN 008 | CRF 02_AG | A1/02_AG* | CRF 02_AG |  |
| SN 009 | 09_cpx, A1, G* | ? | CRF 02_AG |  |
| SN 010 | A (A1) | A1/02_AG* | CRF 02_AG |  |
| SN 011 | CRF 02_AG | 02_AG | CRF 02_AG |  |
| SN 012 | CRF 02_AG | A1/02_AG* | CRF 02_AG |  |
| SN 013 | A (02_AG) | A1/02_AG* | CRF 02_AG |  |
| SN 014 | CRF 02_AG | 02_AG | CRF 02_AG |  |
| SN 015 | A (02_AG) | 02_AG | CRF 02_AG |  |
| SN 016 | CRF 02_AG | 02_AG | CRF 02_AG |  |
| SN 017 | CRF 02_AG | 02_AG | CRF 02_AG |  |
| SN 018 | A (02_AG) | 02_AG | CRF 02_AG |  |
| SN 019 | CRF 02_AG | 02_AG | CRF 02_AG |  |
| SN 020 | A (02_AG) | 02_AG | CRF 02_AG |  |
| SN 021 | A (02_AG) | 02_AG | CRF 02_AG |  |
| SN 022 | A (02_AG) | A1/02_AG* | CRF 02_AG |  |
| SN 023 | CRF 02_AG | ? | CRF 02_AG |  |
| SN 024 | CRF 02_AG | ? | CRF 02_AG |  |
| SN 025 | A (02_AG) | 02_AG | CRF 02_AG |  |
| SN 026 | A (02_AG) | A1/02_AG* | CRF 02_AG |  |
| SN 027 | CRF 02_AG | A1/02_AG* | CRF 02_AG |  |
| SN 028 | A (02_AG) | 02_AG | CRF 02_AG |  |
| SN 029 | A (02_AG) | 02_AG | CRF 02_AG |  |
| SN 030 | CRF 02_AG | 02_AG | CRF 02_AG |  |
| SN 031 | A (A1) | A1 | A3 |  |
| SN 032 | CRF 02_AG | 02_AG | CRF 02_AG |  |
| SN 033 | CRF 02_AG | 02_AG | CRF 02_AG |  |
| SN 034 | CRF 02_AG | 02_AG | CRF 02_AG |  |
| SN 035 | CRF 02_AG | 02_AG | CRF 02_AG |  |
| SN 036 | A (02_AG) | 02_AG | CRF 02_AG |  |
| SN 037 | CRF 02_AG | 02_AG | CRF 02_AG |  |
| SN 038 | A (02_AG) | 02_AG | CRF 02_AG |  |
| SN 039 | CRF 02_AG | A1/02_AG* | CRF 02_AG |  |
| SN 040 | CRF 02_AG | 02_AG | CRF 02_AG |  |
| SN 041 | G* | 06_cpx | CRF 06_cpx |  |
| SN 042 | CRF 02_AG | 02_AG | CRF 02_AG |  |
| SN 043 | A (A1) | 01_AE | CRF 22_01A1 |  |
| SN 044 | G, A1* | ? | CRF 06_cpx |  |
| SN 045 | CRF 02_AG | 02_AG | CRF 02_AG |  |
| SN 046 | A (A1) | A1 | A3 |  |
| SN 047 | CRF 02_AG | 02_AG | CRF 02_AG |  |
| SN 048 | A (A1) | A1 | A3 |  |
| SN 049 | CRF 02_AG | 02_AG | CRF 02_AG |  |
| SN 050 | CRF 02_AG | 02_AG | CRF 02_AG |  |
| SN 051 | CRF 02_AG | 02_AG | CRF 02_AG |  |
| SN 052 | CRF 02_AG | 02_AG | CRF 02_AG |  |
| SN 053 | A (A1) | A1 | A3 |  |
| SN 054 | CRF 02_AG | A1 (02_AG) | CRF 02_AG |  |
| SN 055 | A (02_AG) | A1 (02_AG) | CRF 02_AG |  |
| SN 056 | G | G | G |  |
| SN 057 | CRF 02_AG | A1 (02_AG) | CRF 02_AG |  |
| SN 058 | A (02_AG) | 02_AG | CRF 02_AG |  |
| SN 059 | CRF 02_AG | A1 (02_AG) | CRF 02_AG |  |
| SN 060 | D | D | D |  |
| SN 061 | A (02_AG) | A1 (02_AG) | CRF 02_AG |  |
| SN 062 | A (02_AG) | 02_AG | CRF 02_AG |  |
| SN 063 | CRF 02_AG | ? | CRF 02_AG |  |
| SN 064 | A (02_AG) | 02_AG | CRF 02_AG |  |
| SN 065 | A (A1) | ? | A3 |  |
| SN 066 | CRF 02_AG | ? | CRF 02_AG |  |
| SN 067 | CRF 02_AG | ? | CRF 02_AG |  |
| SN 068 | G | G | G |  |
| SN 069 | 02_AG, A1 | ? | CRF 02_AG |  |
| SN 070 | CRF 02_AG | ? | CRF 02_AG |  |
| SN 071 | CRF 02_AG | ? | CRF 02_AG |  |
| SN 072 | D | D | D |  |
| SN 073 | CRF 02_AG | ? | CRF 02_AG |  |
| SN 074 | A1 | ? | CRF 02_AG |  |
| SN 075 | D | D | D |  |
| SN 076 | A (A1) | A1 | A3 |  |
| SN 077 | CRF 02_AG | 02_AG | CRF 02_AG |  |
| SN 078 | CRF 02_AG | A1 (02_AG) | CRF 02_AG |  |
| SN 079 | A (A1) | A1 | A3 |  |
| SN 080 | CRF 02_AG | A1 (02_AG) | CRF 02_AG |  |
| SN 081 | CRF 02_AG | A1 (02_AG) | CRF 02_AG |  |
| SN 082 | A (02_AG) | A1 (02_AG) | CRF 02_AG |  |
| SN 084 | CRF 02_AG | ? | CRF 02_AG |  |
| SN 085 | CRF 02_AG | ? | CRF 02_AG |  |
| SN 086 | A (02_AG) | A1 (02_AG) | CRF 02_AG |  |
| SN 087 | CRF 02_AG | A1 (02_AG) | CRF 02_AG |  |
| SN 088 | A (02_AG) | 02_AG | A3 |  |
| SN 089 | A (A1) | A1 (02_AG) | CRF 02_AG |  |
| SN 090 | A (02_AG) | A1 (02_AG) | CRF 02_AG |  |
| SN 091 | B | B | B |  |
| SN 092 | A1, G | A1, G | A1, G |  |
| SN 093 | A1, G | ? | CRF 02_AG |  |
| SN 094 | A (02_AG) | 02_AG | CRF 02_AG |  |
| SN 096 | A1 | ? | A3 |  |

*Low Confidence or bootstrap (<70%); ^?^Sequence could not be subtyped by online tool; Mphy: Molecular phylogeny

**Supplementary Table 2: East African samples *gag–protease* subtype assignment**

| SAMPLE ID | REGA | COMET | Mphy |  |
| --- | --- | --- | --- | --- |
|  |  |  |  |  |
| PC 002 | A (A1) | A1 | A1 |  |
| PC 003 | A1, C | A1C* | A1C |  |
| PC 004 | A (A1) | A1 | A1 |  |
| PC 005 | A (A1) | A1 | A1 |  |
| PC 006 | A (A1) | A1 | A1 |  |
| PC 008 | A (A1) | A1 | A1 |  |
| PC 009 | A (A1) | A1 | A1 |  |
| PC 010 | A (A1) | A1 | A1 |  |
| PC 011 | A1 | A1 | A1 |  |
| PC 012 | C, A1 | ? | A1C |  |
| PC 013 | A1, C | A1C* | A1C |  |
| PC 014 | A (A1) | A1 | A1 |  |
| PC 015 | D, A1 | A1D* | A1D |  |
| PC 016 | A (A1) | A1 | A1 |  |
| PC 017 | A (A1) | ? | A1 |  |
| PC 018 | A (A1) | A1 | A1 |  |
| PC 020 | A (A1) | A1 | A1 |  |
| PC 022 | D | D | D |  |
| PC 023 | D | D | D |  |
| PC 025 | D | D | D |  |
| PC 026 | A1, D | ? | A1D |  |
| PC 027 | A (A1) | A1 | A1 |  |
| PC 028 | A (A1) | A1 | A1 |  |
| PC 029 | A (A1) | A1 | A1 |  |
| PC 030 | A (A1) | A1 | A1 |  |
| PC 031 | A (A1) | A1 | A1 |  |
| PC 032 | A (A1) | A1 | A1 |  |
| PC 033 | A (A1) | A1 | A1 |  |
| PC 034 | A (A1) | A1 | A1 |  |
| PC 036 | C, A1 | A1C* | A1C |  |
| PC 039 | A1, D | ? | A1D |  |
| PC 040 | A (A1) | A1 | A1 |  |
| PC 041 | A (A1) | A1 | A1 |  |
| PC 042 | A (A1) | A1 | A1 |  |
| PC 043 | A (A1) | A1 | A1 |  |
| PC 046 | C, A1 | A1C* | A1C |  |
| PC 047 | A (A1) | A1 | A1 |  |
| PC 049 | D | D | D |  |
| PC 050 | A (A1) | A1 | A1 |  |
| PC 051 | A1, D | ? | A1D |  |
| PC 053 | A1, D | ? | A1D |  |
| PC 054 | D | D | D |  |
| PC 055 | A (A1) | A1 | A1 |  |
| PC 056 | A1D | A1D | A1D |  |
| PC 057 | A (A1) | A1 | A1 |  |
| PC 058 | D | D | D |  |
| PC 060 | D | D | D |  |
| PC 061 | A (A1), | A1 | A1D |  |
| PC 062 | A (A1)-like | A1D* | A1D |  |
| PC 063 | A1 | A1 | A1 |  |
| PC 066 | D | D | D |  |
| PC 067 | A (A1) | A1C* | A1 |  |
| PC 068 | D | D | D |  |
| PC 070 | A1 | A1 | A1 |  |
| PC 071 | D, A1 | A1D* | D |  |
| PC 076 | A (A1) | A1 | A1 |  |
| PC 077 | A (A1) | A1 | A1 |  |
| PC 078 | A1 | A1 | A1 |  |
| PC 079 | A1, H | ? | A1D |  |
| PC 080 | A1 | A1 | A1 |  |
| PC 081 | A (A1) | A1 | A1 |  |
| PC 083 | A (A1) | A1 | A1 |  |
| PC 087 | A (A1) | A1 | A1 |  |
| PC 088 | A (A1) | A1 | A1 |  |
| PC 089 | A (A1) | A1 | A1 |  |
| PC 092 | A (A1) | A1 | A1 |  |
| PC 093 | A (A1) | A1 | A1 |  |
| PC 095 | D | D | D |  |
| PC 096 | A1, D | A1D* | A1D |  |
| PC 097 | A (A1) | A1 | A1 |  |
| PC 098 | A (A1) | A1 | A1 |  |
| PC 099 | D | A1 | D |  |
| PC 100 | A1 | A1 | A1 |  |
| PC 101 | A (A1) | A1 | A1 |  |
| PC 104 | A (A1) | A1 | A1 |  |
| PC 105 | A (A1) | A1 | A1 |  |
| PC 106 | A (A1) | A1 | A1 |  |
| PC 107 | A (A1) | A1 | A1 |  |
| PC 109 | A (A1) | A1 | A1 |  |
| PC 112 | A (A1) | A1 | A1 |  |
| PC 113 | A (A1) | A1 | A1 |  |
| PC 114 | D | A1D* | A1D |  |
| PC 116 | A (A1) | A1 | A1 |  |
| PC 117 | A (A1) | A1 | A1 |  |
| PC 118 | A (A1) | A1 | A1 |  |
| PC 121 | A1 | A1 | A1 |  |
| PC 122 | A (A1) | A1 | A1 |  |
| PC 123 | D | D | D |  |
| PC 125 | D | D | D |  |
| PC 128 | A (A1) | A1 | A1 |  |
| PC 129 | A (A1) | A1 | A1 |  |
| PC 132 | A (A1) | A1 | A1 |  |
| PC 137 | A (A1) | A1 | A1 |  |
| PC 141 | A (A1) | A1 | A1 |  |
| PC 142 | A1, D | ? | A1D |  |
| PC 143 | A (A1) | A1 | A1 |  |
| PC 144 | A (A1) | A1 | A1 |  |
| PC 148 | A1 | A1 | A1 |  |
| PC 149 | D | D | D |  |
| PC 150 | A1, D | A1D* | A1D |  |
| PC 151 | A (A1) | A1 | A1 |  |
| PC 154 | A (A1) | A1 | A1 |  |
| PC 155 | A1 | A1 | A1 |  |
| PC 158 | A (A1) | A1 | A1 |  |
| PC 159 | A (A1) | A1 | A1 |  |
| PC 161 | A (A1) | A1 | A1 |  |
| PC 162 | A (A1) | A1 | A1 |  |
| PC 163 | D | D1 | D |  |
| PC 164 | A (A1) | A1 | A1 |  |
| PC 165 | D, A1 | A1D* | D |  |
| PC 166 | D | D | D |  |
| PC 167 | D, A1 | A1D* | A1D |  |
| PC 168 | D | D | D |  |
| PC 170 | D | D | D |  |
| PC 171 | D | D | D |  |
| PC 172 | D | D | D |  |
| PC 173 | A (A1) | A1 | A1 |  |
| PC 174 | A (A1) | A1 | A1 |  |
| PC 175 | A1, D | A1D* | A1D |  |
| PC 176 | A (A1) | A1 | A1 |  |
| PC 177 | A (A1) | A1 | A1 |  |
| PC 178 | D | D | D |  |
| PC 179 | A (A1) | A1 | A1 |  |
| PC 181 | D | D | D |  |
| PC 182 | D | D | D |  |
| PC 183 | D | D | D |  |
| PC 185 | D | D | D |  |
| PC 186 | D | D | D |  |
| PC 187 | D | D | D |  |
| PC 188 | A (A1) | A1 | A1 |  |
| PC 190 | D | D | D |  |
| PC 191 | D | D | D |  |
| PC 192 | D | D | D |  |
| PC 193 | D | D | D |  |
| PC 194 | A (A1) | A1 | A1 |  |
| PC 195 | A (A1) | A1 | A1 |  |
| PC 196 | D | D | D |  |
| PC 197 | A (A1) | A1 | A1 |  |
| PC 198 | A (A1) | A1 | A1 |  |
| PC 199 | A (A1) | A1 | A1 |  |
| PC 200 | A (A1) | A1 | A1 |  |
| PC 201 | A (A1) | A1 | A1 |  |
| PC 202 | A (A1) | A1 | A1 |  |
| PC 203 | A (A1) | A1 | A1 |  |
| PC 204 | A (A1) | A1 | A1 |  |
| PC 205 | A (A1) | A1 | A1 |  |
| PC 206 | A (A1) | A1 | A1 |  |
| PC 213 | D | D | D |  |
| PC 217 | A (A1) | A1 | A1 |  |
| PC 222 | A (A1) | A1 | A1 |  |
| PC 223 | D, A1 | ? | A1D |  |
| PC 224 | A (A1) | A1 | A1 |  |
| PC 225 | A (A1) | A1 | A1 |  |
| PC 227 | A1D | A1D | A1D |  |
| PC 229 | A (A1) | A1 | A1 |  |
| PC 230 | A (A1) | A1 | A1 |  |
| PC 232 | A (A1) | A1 | A1 |  |
| PC 233 | A (A1) | A1 | A1 |  |
| PC 235 | A (A1) | A1 | A1 |  |
| PC 239 | A1, D | ? | A1 |  |

*Low Confidence or bootstrap (<70%)

^?^Sequence could not be subtyped by online tool;

Mphy: Molecular phylogeny

Supplementary Figure 1


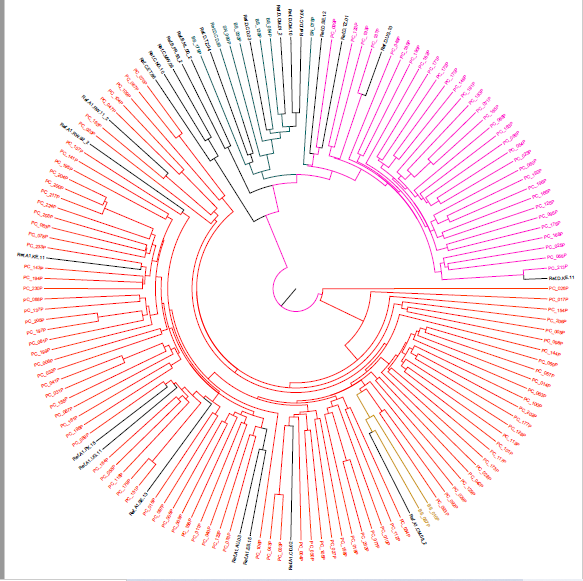


0.03

**Supplementary Figure 1:** Maximum likelihood tree showing common subtypes A1 and D in West and East Africa with clustering indicative of region-specific subtype evolution.

| West Africa | A1 | D |
| --- | --- | --- |
| East Africa | A1 | D |
